# Supplementary material for: Novel methods for estimating the instantaneous and overall COVID-19 case fatality risk among care home residents in England
Source: PLoS Comput Biol. 2022 Oct 24;18(10):e1010554. doi: 10.1371/journal.pcbi.1010554 (PMC9632866; doi:10.1371/journal.pcbi.1010554)
Supplement: S1 Text — (PDF) [file pcbi.1010554.s001.pdf]

S2: Supplementary methods 1: Novel methods for estimating the instantaneous and overall COVID-19 case fatality risk among care home residents in England

Christopher E. Overton[1,2,3,11], Luke Webb[1], Uma Datta[4], Mike Fursman[4], Jo Hardstaff[6], Iina Hiironen[6], Karthik Paranthaman[6], Heather Riley[1], James Sedgwick[6], Julia Verne[7,8], Steve Willner[7], Lorenzo Pellis[1,3,10], and Ian Hall[1,2,3,9,10]

[1] *Department of Mathematics, University of Manchester, UK.*
[2] *Clinical data science unit, Manchester University NHS Foundation Trust, UK.*
[3] *Joint UNIversities Pandemic and Epidemiological Research, https://maths.org/juniper/.*
[4] *Care Quality Commission*
[6] *Field Service, National Infection Service, Public Health England, UK.*
[7] *Adult Social Care Team, Public Health England, UK.*
[8] *Office for Health Improvement and Disparities, Department of Health and Social Care, UK.*
[9] *Emergency Preparedness, Health Protection Division, Public Health England, UK.*
[10] *Alan Turing Institute, UK.*
[11] *Data, Analytics and Surveillance, UK Health Security Agency, UK.*

October 1, 2022

1 Data sources

1.1 Data from Public Health England

1.1.1 Positive cases and deaths among residents in care homes

We used a de-duplicated, non-identifiable line list of positive SARS-CoV-2 tests among care home residents derived from PHE's national database for laboratory results (Second Generation Surveillance System) and enriched by other data sources. This dataset includes date of positive SARS-CoV-2 test, date of death, age, care home post code

1.2 Data from the Care Quality Commission (CQC)

1.2.1 Care home locations

The Care Quality Commission (CQC) publish a monthly list of active locations, registered with the CQC. This has a care home filter that was used to only select care homes. These include nursing homes, homes for less able people and young persons' homes and information such as names, addresses, postcodes and local authority. This was updated each month to be as accurate as possible.

1.2.2 Care home deaths

Individual care homes report all mortality to the CQC. As part of this, the care homes identify whether COVID-19 contributed to the cause of death. This could be as recorded on the death certificate or determined by the care home staff. The possible categories are: Confirmed, Suspected, non-COVID, and unknown. For the purposes of our analysis, we filter the data to only include "Confirmed" COVID-19 deaths. This dataset includes: care home ID, type of care, date of report, number of deaths reported, COVID death type, and place of death. Rather than reported individual deaths, this data records the number of deaths reported

by the given location on the date of report. The place of death includes options such as "Hospital" and "Reporting location" (i.e. care home). For our analysis, we included all places of death. We removed "Domicillary care service" from the type of care field, since we are only interested in care homes rather than other types of care provided.

1.3 Combining datasets

From linking the PHE linelist to CQC care home location data via care home postcode, the following variables were obtained: age, specimen date, date of death, postcode, and type of care. Although the PHE data had already been linked to care homes, we needed to match to the care home locations data to extract the type of care provided. The resulting data were filtered to only include individuals over 65, to ensure the majority of individuals were care home residents rather than staff.

Again using postcode matching, we then linked the CQC care home reported deaths data to daily aggregated deaths and cases per care home from the PHE data. This generated a data set containing the number of cases per day, number of deaths per day, and number of deaths reported to CQC per day, for each individual care home.
