# Supplementary material for: Novel methods for estimating the instantaneous and overall COVID-19 case fatality risk among care home residents in England
Source: PLoS Comput Biol. 2022 Oct 24;18(10):e1010554. doi: 10.1371/journal.pcbi.1010554 (PMC9632866; doi:10.1371/journal.pcbi.1010554)
Supplement: S2 Text — (PDF) [file pcbi.1010554.s002.pdf]

S2: Supplementary methods 1: Novel methods for estimating the instantaneous and overall COVID-19 case fatality risk among care home residents in England

Christopher E. Overton[1,2,3,11], Luke Webb[1], Uma Datta[4], Mike Fursman[4], Jo Hardstaff[6], Iina Hiironen[6], Karthik Paranthaman[6], Heather Riley[1], James Sedgwick[6], Julia Verne[7,8], Steve Willner[7], Lorenzo Pellis[1,3,10], and Ian Hall[1,2,3,9,10]

[1] *Department of Mathematics, University of Manchester, UK.*
[2] *Clinical data science unit, Manchester University NHS Foundation Trust, UK.*
[3] *Joint UNIversities Pandemic and Epidemiological Research, https://maths.org/juniper/.*
[4] *Care Quality Commission*
[6] *Field Service, National Infection Service, Public Health England, UK.*
[7] *Adult Social Care Team, Public Health England, UK.*
[8] *Office for Health Improvement and Disparities, Department of Health and Social Care, UK.*
[9] *Emergency Preparedness, Health Protection Division, Public Health England, UK.*
[10] *Alan Turing Institute, UK.*
[11] *Data, Analytics and Surveillance, UK Health Security Agency, UK.*

October 1, 2022

1 Estimating the delay distribution from testing positive to death

The time from testing positive to dying is given by a random variable. Finding the distribution of this random variable is essential when estimating the case fatality risk. To estimate the delay distribution, we need to look at the time between the two events involved, in this case testing positive and death. Events are only observed if they occur before the final sampling date. Therefore, if the first event occurs near to the end of the sampling window, it will only be observed if the delay to the second event is short. This truncation causes an over-expression of short delays towards the end of the sampling window, which is exacerbated by exponential growth during a growing epidemic. Therefore, we need to account for truncation within our estimation framework.

To fit the data, we use maximum likelihood estimation. However, we do not observe the delay directly, but rather the times of the two events. Therefore, we need to construct a likelihood function for observing these events. Following [1], we construct the conditional density function for observing the second event given the time of the first event and given that the second event occurs before date T. That is, we are interested in the conditional density function

$$L(D = d|T = t, D \leq \tau) = \frac{g(t)f(d-t)}{\int_0^{\tau-t} g(t)f(x)\mathrm{d}x} = \frac{f(d-t)}{\int_0^{\tau-t} f(x)\mathrm{d}x}, \tag{1}$$

where f is the probability distribution function of the delay from testing positive to dying, g is the probability distribution function of testing positive on each day, T and D denote random times of testing positive and dying, respectively, and small letters their realisations, and τ is the truncation date.

Using this truncation-corrected method and a Gamma distribution to fit the delay distribution, we can estimate the delay distribution. Since the delay may not be constant over time, we disaggregate the sample based on the month when an individual tests positive. This allows us to estimate specific delay distributions for each month, capturing temporal changes in the distribution.

Through this, we obtain gamma distributed delay distributions with means and standard deviations as shown in Table A1.

Table A1: Gamma distributed delays from testing positive to death within 28 days. In the CFR estimation, we only use the point estimates for the mean and standard deviation to parameterise the delay distribution. Here we report 95% confidence intervals about the mean (generated through parametric bootstrapping) to illustrate uncertainty in the estimates.

**Month**	**Mean**	**Standard deviation**	**Sample size**
**Overall**	10.52 (10.43, 10.61)	7.59	29690
**March 2020**	8.15 (7.83, 8.49)	6.51	1380
**April 2020**	7.86 (7.69, 8.00)	6.63	6728
**May 2020**	9.58 (9.27, 9.87)	7.45	2465
**June 2020**	11.48 (10.73, 12.22)	7.86	368
**July 2020**	11.37 (9.54, 13.49)	8.84	79
**August 2020**	12.33 (10.29, 14.26)	8.38	63
**September 2020**	12.58 (11.65, 13.45)	8.46	332
**October**	12.20 (11.84, 12.57)	7.39	1517
**November 2020**	11.99 (11.73, 12.26)	7.45	2909
**December 2020**	11.80 (11.58, 12.00)	7.14	4323
**January 2021**	11.47 (11.31, 11.62)	7.14	7447
**February 2021**	11.72 (11.23, 12.21)	7.83	1076
**March 2021**	12.22 (11.07, 13.38)	7.96	175
**April 2021**	11.71 (8.93, 14.29)	8.22	34
**May 2021**	NA	NA	17
**June 2021**	11.13 (8.99, 13.33)	8.08	52
**July 2021**	12.46 (11.49, 13.55)	7.53	206
**August 2021**	12.98 (12.14, 13.81)	8.38	380

References

[1] J. Sun. Empirical estimation of a distribution function with truncated and doubly interval-censored data and its application to AIDS studies. *Biometrics*, pages 1096–1104, 1995.
