# Supplementary material for: Novel methods for estimating the instantaneous and overall COVID-19 case fatality risk among care home residents in England
Source: PLoS Comput Biol. 2022 Oct 24;18(10):e1010554. doi: 10.1371/journal.pcbi.1010554 (PMC9632866; doi:10.1371/journal.pcbi.1010554)
Supplement: S3 Text — (PDF) [file pcbi.1010554.s003.pdf]

S2: Supplementary methods 1: Novel methods for estimating the instantaneous and overall COVID-19 case fatality risk among care home residents in England

Christopher E. Overton[1,2,3,11], Luke Webb[1], Uma Datta[4], Mike Fursman[4], Jo Hardstaff[6], Iina Hiironen[6], Karthik Paranthaman[6], Heather Riley[1], James Sedgwick[6], Julia Verne[7,8], Steve Willner[7], Lorenzo Pellis[1,3,10], and Ian Hall[1,2,3,9,10]

[1] *Department of Mathematics, University of Manchester, UK.*
[2] *Clinical data science unit, Manchester University NHS Foundation Trust, UK.*
[3] *Joint UNIversities Pandemic and Epidemiological Research, https://maths.org/juniper/.*
[4] *Care Quality Commission*
[6] *Field Service, National Infection Service, Public Health England, UK.*
[7] *Adult Social Care Team, Public Health England, UK.*
[8] *Office for Health Improvement and Disparities, Department of Health and Social Care, UK.*
[9] *Emergency Preparedness, Health Protection Division, Public Health England, UK.*
[10] *Alan Turing Institute, UK.*
[11] *Data, Analytics and Surveillance, UK Health Security Agency, UK.*

October 1, 2022

1 Derivation of the existing backward methods

The daily-instantaneous backward method CFR is defined as

$$CFR_b(t) = \frac{d(t)}{\tilde{C}(t)}, \tag{1}$$

where $\tilde{C}(t)$ is the adjusted number of cases and $d(t)$ is the number of deaths on day t.

To calculate $\tilde{C}(t)$, we need to look at the number of cases on each previous day and scale this by the proportion of deaths that would occur on day t given that the first positive test was on this day and that the individual goes on to die. That is

$$\tilde{C}(t) = \sum_{x=0}^{t} C(x)P(D = t|I = x \cap 1_D = 1) = \sum_{x=0}^{t} C(x)g(t-x|x), \tag{2}$$

where 1_D is an indicator function on whether the individual goes on to die. Therefore, we have

$$CFR_b(t) = \frac{d(t)}{\sum\limits_{x=0}^{t} C(x)g(t-x|x)}. \tag{3}$$

To generate an overall CFR using the backwards approach, we need to see what proportion of cases ended in deaths up to the given date. The overall backward CFR on day t is given by the number of deaths up to and including day t by the expected number of cases that could have ended in death on or before day t. That is,

$$oCFR_b(t) = \frac{\sum\limits_{x=0}^{t} d(x)}{\sum\limits_{x=0}^{t} c(x)P(D \le t|I = x \cap 1_D = 1)} = \frac{\sum\limits_{x=0}^{t} d(x)}{\sum\limits_{x=0}^{t} c(x)F_\theta(t-x|x))}, \tag{4}$$

where $F_\theta(\cdot)$ is the cumulative distribution function of the testing to death delay.
