# Supplementary material for: Novel methods for estimating the instantaneous and overall COVID-19 case fatality risk among care home residents in England
Source: PLoS Comput Biol. 2022 Oct 24;18(10):e1010554. doi: 10.1371/journal.pcbi.1010554 (PMC9632866; doi:10.1371/journal.pcbi.1010554)
Supplement: S4 Text — (PDF) [file pcbi.1010554.s004.pdf]

S4: Supplementary methods 3

Novel methods for estimating the instantaneous and overall COVID-19 case fatality risk among care home residents in England

Christopher E. Overton, Luke Webb, Uma Datta, Mike Fursman, Jo Hardstaff, Iina Hiironen, Karthik Paranthaman, Heather Riley, James Sedgwick, Julia Verne, Steve Willner, Lorenzo Pellis, and Ian Hall

Supplementary Methods

Risk ratio

In this supplementary material, we quantify the difference between CFR across different groups using the risk ratio. The analysis shown here is all based on the backward method for calculating the CFR. Therefore, we assume deaths are given by observed deaths, $d(t)$, and construct an adjusted denominator, $\tilde{C}(t)$, for cases that could die on day t. For each group, we take the number of exposed individuals to be $\tilde{C}(t)$ and number of outcomes among exposed individuals to be $d(t)$. From these, we calculate the risk ratio in R [1] using the riskratio function from the epitools package [2]. These risk ratios are used to complement the comparisons provided in the main text.

Relative error

To score the accuracy of the CFR methods at approximating the cohort CFR, we calculate the relative error between the cohort CFR and the approximation. That is,

$$\text{relative\_error} = \frac{observed - predicted}{observed}.$$

Supplementary References

[1] R Core Team (2020). R: A language and environment for statistical computing. R Foundation for Statistical Computing, Vienna, Austria. URL: https://www.R-project.org/.

[2] Tomas J. Aragon (2020). epitools: Epidemiology Tools. R package version 0.5-10.1. URL: https://CRAN.R-project.org/package=epitools
