# Supplementary material for: Novel methods for estimating the instantaneous and overall COVID-19 case fatality risk among care home residents in England
Source: PLoS Comput Biol. 2022 Oct 24;18(10):e1010554. doi: 10.1371/journal.pcbi.1010554 (PMC9632866; doi:10.1371/journal.pcbi.1010554)
Supplement: S5 Text — (PDF) [file pcbi.1010554.s005.pdf]

S5: Supplementary results

Novel methods for estimating the instantaneous and overall COVID-19 case fatality risk among care home residents in England

Christopher E. Overton, Luke Webb, Uma Datta, Mike Fursman, Jo Hardstaff, Iina Hiironen, Karthik Paranthaman, Heather Riley, James Sedgwick, Julia Verne, Steve Willner, Lorenzo Pellis, and Ian Hall

Supplementary Results

Age

From the risk ratios by age group (Fig A1), the 65 to 75 age group has a lower CFR than the 75 to 85 age group and the 85+ age group. The 75 to 85 age group has a lower CFR than the 85+ age group.

Care type

From the risk ratios by care type (Fig A2), the picture is unclear. The overall CFR has been higher in care homes with nursing relative to those without. However, the instantaneous CFR rarely has a significant difference. The daily instantaneous CFR has no significant difference. The weekly instantaneous CFR occasionally has a significant difference. However, despite being mostly insignificant, the risk ratio central estimate shows increased risk with nursing for most of the time series, which is what leads to the overall CFR showing a significantly increased risk ratio in care homes with nursing.

Region

Comparing the risk ratio in each region to the national average using PHE data (Figs A3 and A4), there are very few times when a region has a significantly different risk. The North East and Yorkshire shows occasionally increased risk relative to the national average for a brief period around September 2020. Using the CQC data, the results show a different picture, with all regions except the North East and Yorkshire experiencing periods of significantly increased and decreased risk, relative to the national average. This is likely driven by differences in reporting delays across the care homes in each region, deviating from the national average reporting delay. Similarly to the PHE data, the North East and Yorkshire shows a significantly increased risk, around Summer 2020 and Spring 2021.

Approximation accuracy

Scoring the backward, backward (shifted), and forward approximation methods relative to the cohort CFR as a ground truth (Fig A5) shows the forward method has the best performance, followed by the shifted backward, followed by the backward method. A strongly performing approximation should have relative error approximately normally distributed about zero. This reflects minimal bias to overestimating or underestimating the CFR. The backwards approach shows substantial periods of bias, reflecting the temporal offset between the backwards approximation and the cohort CFR. The backward shifted method has improved performance with fewer periods of bias. However, there is still some slight bias, which is likely driven by changes to the delay distribution over time. Overall accuracy can be measured by the mean relative error. For the forward method, the mean relative error is -0.0132, the backward method mean relative error is -0.0919, and the shifted backward method mean relative error is -0.0487. This verifies that the forward method gives the best approximation to the cohort CFR, followed by shifted backward, followed by backward.

Supplementary Figures

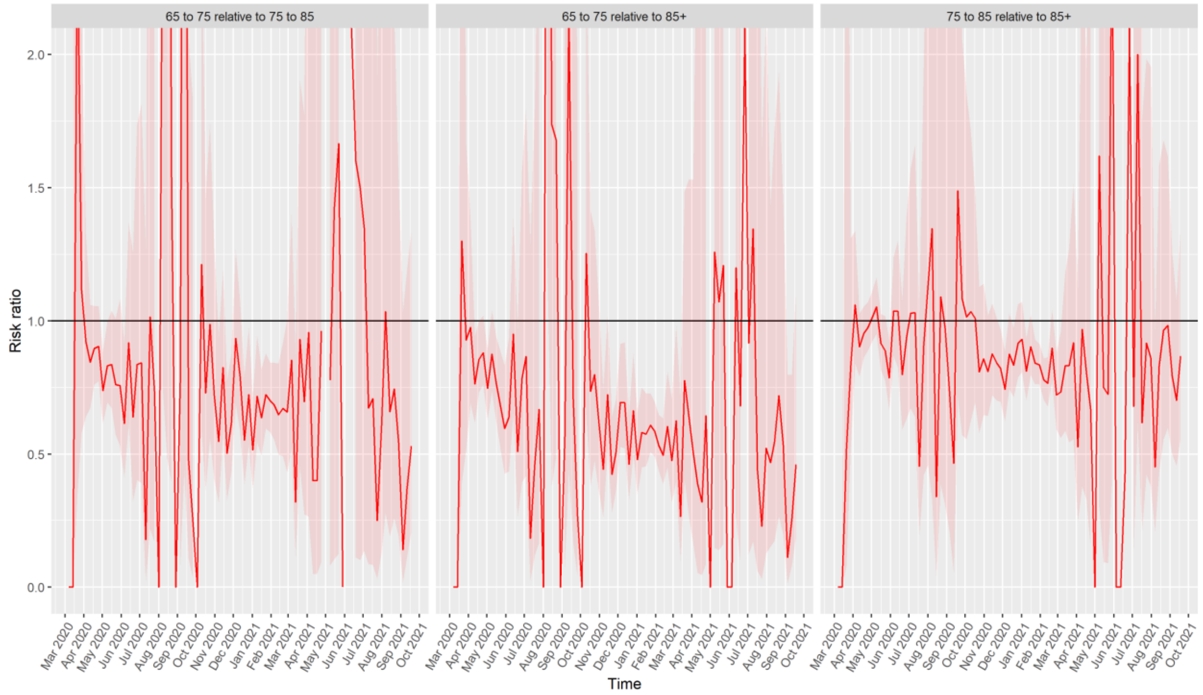

Figure A1: *Case fatality risk ratios over time between the different age groups of care home residents, using the weekly backwards method. The red lines indicate the central estimate, with the red ribbons 95% confidence intervals. The solid line at 1 indicates equal risk.*

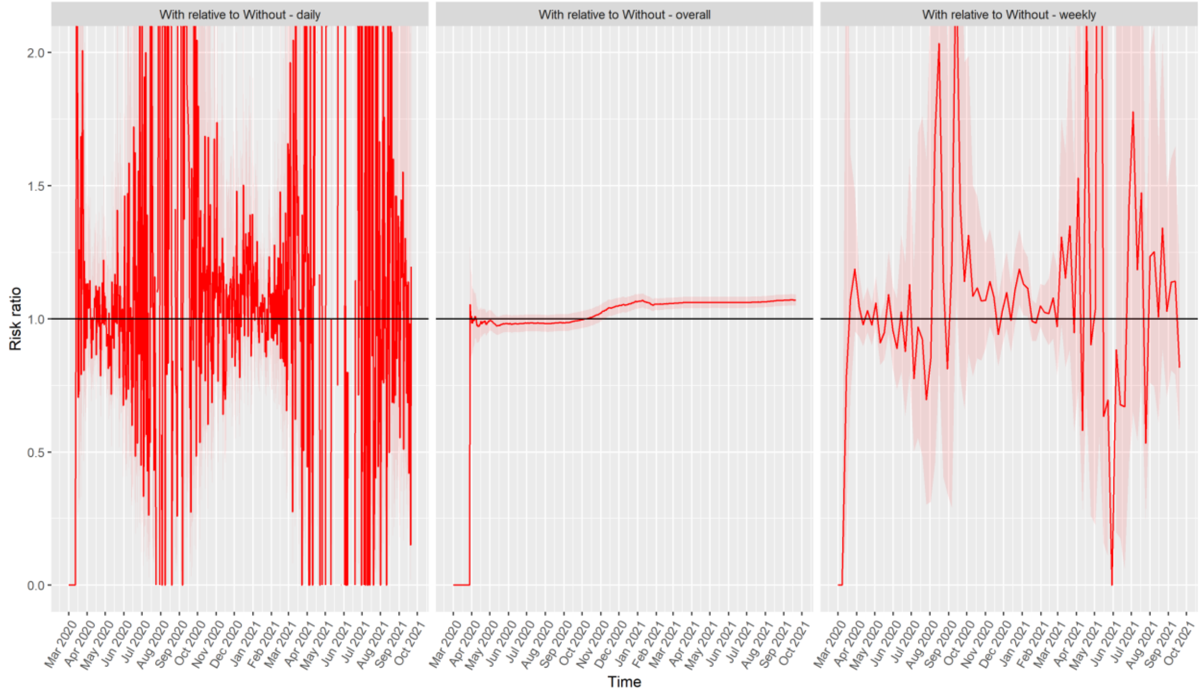

Figure A2: *Case fatality risk ratios over time between the different types of care, for daily (left), weekly (middle) and overall (right) backwards methods. The red lines indicate the central estimate, with the red ribbons 95% confidence intervals. The solid line at 1 indicates equal risk.*

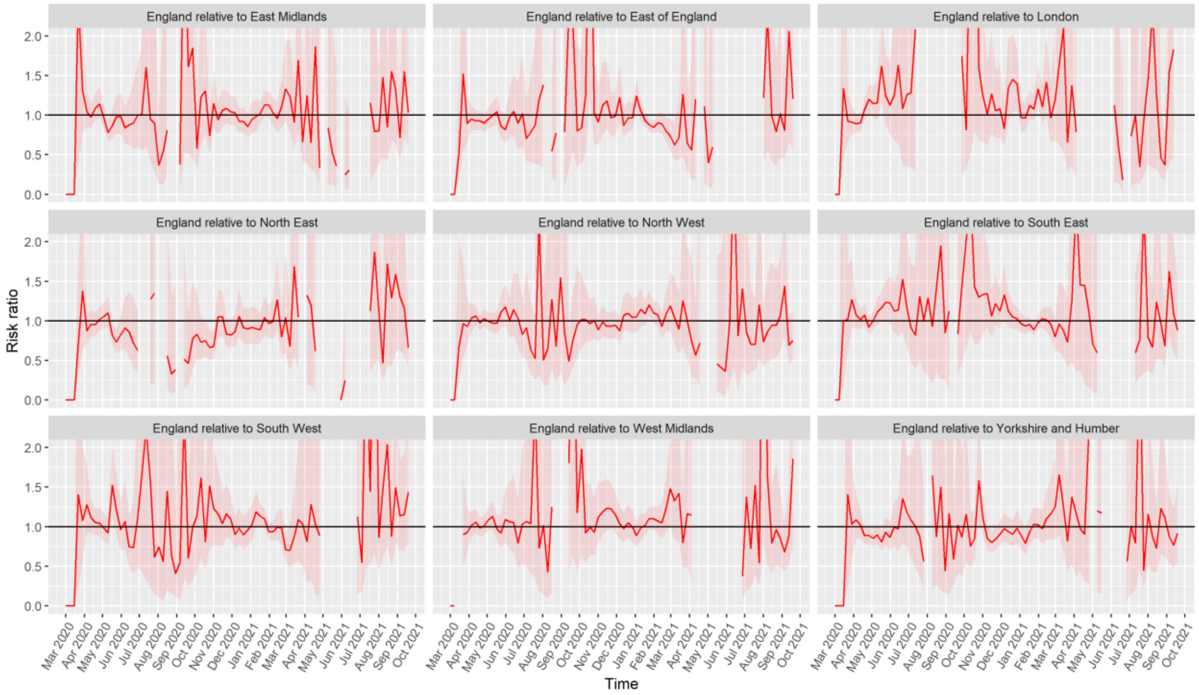

Figure A3: *Case fatality risk ratios over time between different regions and the national trend, using the weekly backwards method and PHE data. The red lines indicate the central estimate, with the red ribbons 95% confidence intervals. The solid line at 1 indicates equal risk.*

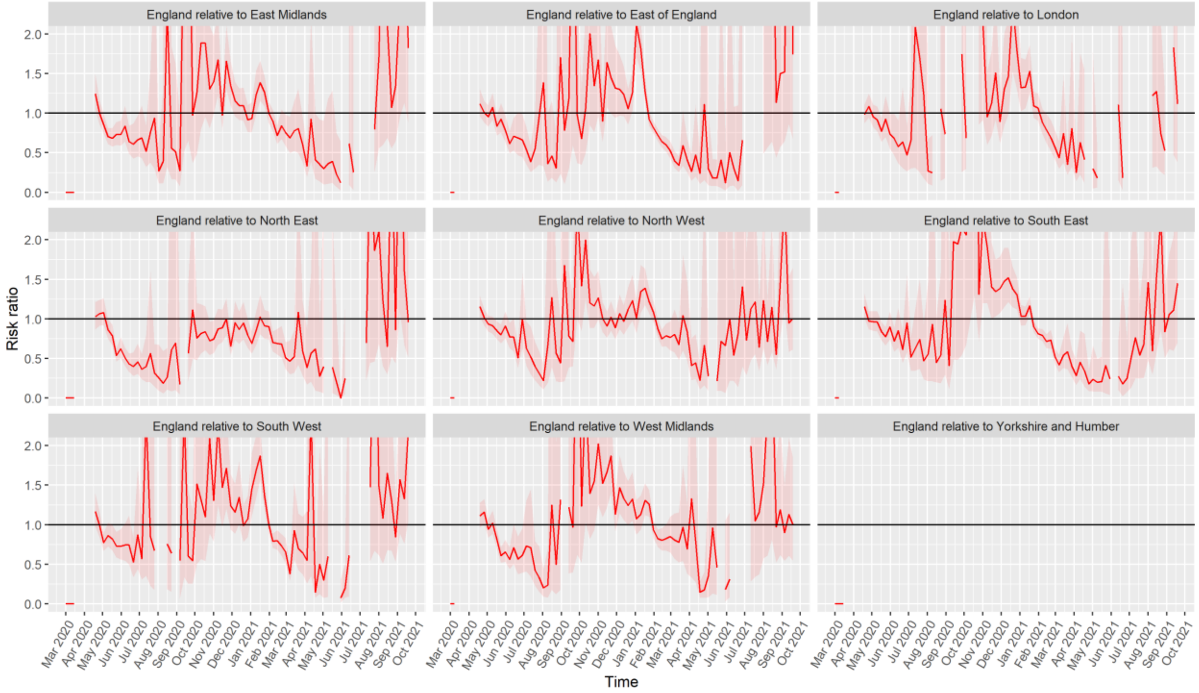

Figure A4: *Case fatality risk ratios over time between different regions and the national trend, using the weekly backwards method and CQC data. The red lines indicate the central estimate, with the red ribbons 95% confidence intervals. The solid line at 1 indicates equal risk.*

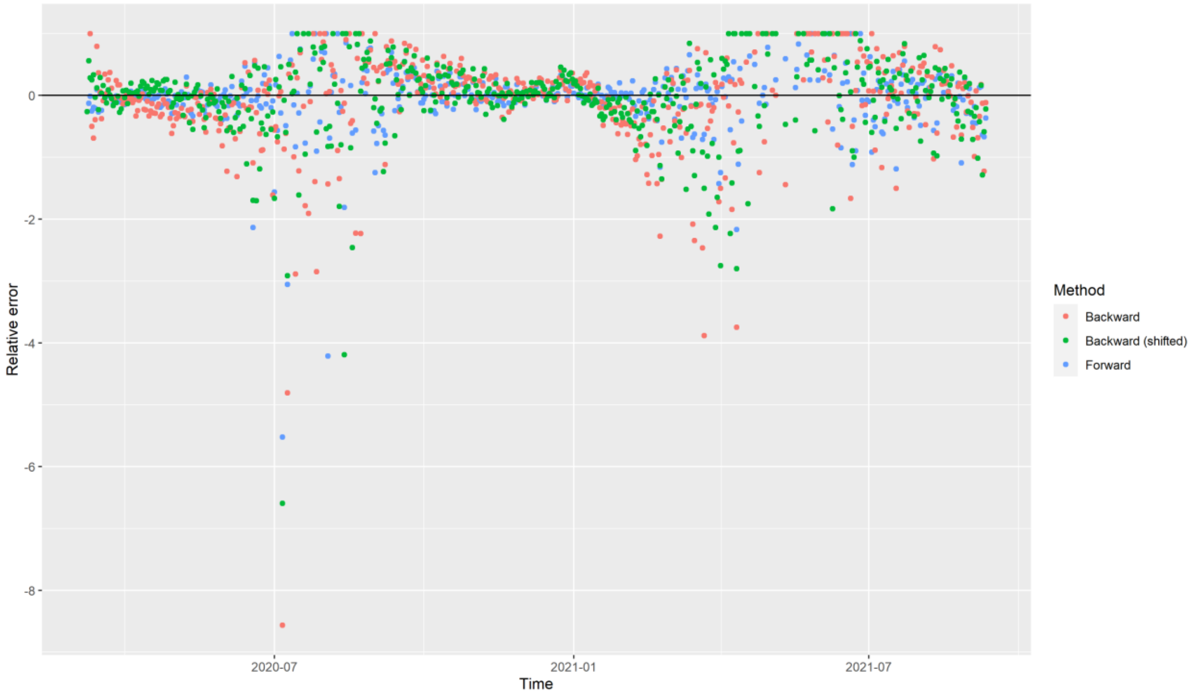

Figure A5: *Relative error between the cohort CFR and the backward (red), backward – shifted (green), and forward (blue) approximation methods. Each point marks the relative error score for the estimates on the corresponding day. The solid line at 0 indicates where the approximation matches the cohort estimates.*
