# Supplementary material for: Novel methods for estimating the instantaneous and overall COVID-19 case fatality risk among care home residents in England
Source: PLoS Comput Biol. 2022 Oct 24;18(10):e1010554. doi: 10.1371/journal.pcbi.1010554 (PMC9632866; doi:10.1371/journal.pcbi.1010554)
Supplement: Attachment — Submitted filename: response_to_reviewers.docx [file pcbi.1010554.s006.docx]

**Comments to the Authors:
Please note here if the review is uploaded as an attachment.**

Reviewer #1: This is a well-written paper describing methods of estimating CFR using either cohort or unlinked time-series datasets. It is in very good shape and could be published as is. However, I do wonder, given that CQC data uses date of report and PHE data uses date of death, is there any benefit to using one rather than the other?

-Thank you for your kind comments. CQC data uses reports from General Practitioners, so may more accurately reflect the total number of deaths where COVID was a contributing factor than the PHE deaths, which counts all deaths within 28 days of a positive test.

Reviewer #2: Dr. Overton and colleagues present a thorough analysis of the CFR of covid-19 among care home residents in England until October 2021. The research question is clear and the introduction provides a good overview of the question. The methods are solid and well explained, and the application is careful and appropriate. The novelty of the approach is not entirely clear, as methods similar to the presented “forward method” have been used in various situations, but the paper provides an interesting application and comparison of different approaches. I also commend the authors for their concern about uncertainty quantification.

-We thank the reviewer for taking time to go through the paper and their kind and helpful comments. We agree that similar methods have been presented before, but the novelty lies within the derivation of all methods within a consistent framework, making it clear how they relate to each other, and the derivation framework allows consistent models to be derived at different temporal scales, such as weekly, whilst still using daily data, enabling accurate adjusting for the time to death delay.

The main weakness of the study is that all comparisons (between the different methods, between age groups, between data sources, between types of care) are only done visually. The authors need to provide a quantification of these differences (with uncertainty) to support their conclusions (e.g. accuracy, sharpness, risk ratios...). There is also a lack of structure in some parts of the results, and interpretations of results need to be moved to the discussion.

-We thank the reviewer for this comment and agree it is an area that needed strengthening. We have restructured the results to move interpretations into the discussion, where relevant. We now include risk ratio comparisons when comparing the CFR across different groups or settings, and have included relative error scoring to evaluate the performance of the different methods. However, the main focus of the narrative remains on the visual comparisons, as these are the strongest ways to highlight the differences between the methods.

Major issues

- The agreement between the different methods is only assessed visually. The authors should provide a quantification of the performance of each method (point estimates and uncertainty) compared to the chosen reference. Various metrics exist, one suggestion could be using accuracy and sharpness as proposed in

Gneiting T, Balabdaoui F, Raftery AE. Probabilistic forecasts, calibration and sharpness. Journal of the Royal Statistical Society: Series B (Statistical Methodology). 2007;69(2):243–268.

-The methods are primarily compared visually as this is the best method to see how they perform, since we are assessing whether they can reproduce similar temporal trends to the “cohort” method as a ground truth. However, we agree that a formal scoring process would be useful to support these analyses. Accuracy and sharpness are powerful for scoring forecasts, and adjusting for their uncertainty, but we do not feel these are appropriate here. The main target for scoring here should be the central estimate, since the methods are focused on getting the central estimates to approximate the cohort CFR, rather than getting the cohort CFR within the confidence intervals. Therefore, we have opted to use relative error scoring to evaluate the performance. These are useful since they can help to identify any direction of bias. The narrative mostly focuses on visual comparison still, as we feel this makes it easier to interpret when the models work and their strengths/weaknesses. We have added this paragraph to the end of the methods comparison section, providing further support to the findings. “To further assess the accuracy of the backward and forward approximation methods, relative to the cohort method, we calculate the relative error (see Supplementary Material for details, Figure S5). The forward method provides the best approximation to the cohort method, with mean relative error equal to -0.0132. The shifted backward comes second, with mean relative error equal to -0.0487. The backward method has the worst performance, with mean relative error equal to -0.0919.”

The same issue appears in comparison of CFR across time, space, age groups, and types of care. Visual comparisons are not sufficient, and the authors should provide a quantification of the differences (e.g. using risk ratios with uncertainty compared to a reference group).

-We have now added risk ratio comparisons when comparing across age groups, care types and space. We have not provided risk ratios when comparing over time, as the visual trends are more powerful here for showing how the CFR is changing. The risk ratios support the conclusions from the visual comparisons of the age, space, and care comparisons. Thank you for this advice for strengthening these comparisons.

- Many of the points discussed in the results have more their place in the discussion, e.g. interpretation of why the CFR was low during the summer 2020 and then increased again in section 4.2.1. The results part could also benefit from more numbered results (e.g. the CFR dropped to around 10% during the summer 2020 before increasing to about 20%…). There are also some repetitions. I feel that the results part would benefit from some level of rewriting, increasing the structure and clarity of the message.

-We have removed most of this discussion from the results section, making the analysis more concise. We have checked that each of these points are thoroughly covered in the discussion. We have added more numerical values to the results sections to improve the flow of the narrative.

Minor points

- A description of the characteristics of the people testing positive in UK care homes that were considered in the paper would be useful, at least regarding age and gender.

We have added a description of the age profile of our cases. “Individuals in English care homes are substantially older, on average, than those in the wider population. Among care home residents who tested positive for COVID-19 in our data, the median age was 86, with 5th percentile equal to 69 and 95th percentile equal to 97. This elderly age profile leads to substantial COVID-19 mortality risk, relative to the wider population.” Thank you for this suggestion, as it highlights the high risk posed to this population. Unfortunately, we do not have data on gender or other characteristics.

- The list of references on methods to estimate the CFR and IFR is relatively light. In particular, I saw no explicit mention of the term “preferential ascertainment of severe cases” that is one of the major biases in CFR estimates (although the author mention this bias implicitely). I would suggest that the authors consider discussing a few additional papers such as

Lipsitch, Marc, et al. "Potential biases in estimating absolute and relative case-fatality risks during outbreaks." PLoS neglected tropical diseases 9.7 (2015): e0003846.

Battegay, Manuel, et al. "2019-novel Coronavirus (2019-nCoV): estimating the case fatality rate–a word of caution." Swiss medical weekly 150.0506 (2020).

Hauser, Anthony, et al. "Estimation of SARS-CoV-2 mortality during the early stages of an epidemic: A modeling study in Hubei, China, and six regions in Europe." PLoS medicine 17.7 (2020): e1003189.

-We have clarified this point in the introduction and added the additional references as suggested: “In particular, preferential ascertainment of severe cases can lead to CFR being biased relative to the IHR”.

- The authors make a very good point about the fact that the CFR may decrease in time because of improvements in care and reduced virulence of variants. I would suggest adding some references on these points.

We thank the reviewer for this suggestion and have added references on both of these points.

- Discussing the instantaneous CFR, the authors state that a daily resolution is too small and results in noisy estimates, and argue in favour of a weekly resolution. I am not entirely convinced by this conclusion, as this entirely depends on the incidence of infection in the population of interest. A weekly resolution may also be too noisy in situations of low incidence or in small populations, and the solution cannot always be aggregating over longer periods of time. A more general solution would be to use multi-level approaches, partially pooling information across time and space to estimate the commonalities and variation of the CFR. Other techniques such as splines or LOESS may also be useful. The authors may consider that this is out of the scope of the curent article, but should at least discuss these points as potential future improvements.

-Thank you for this comment. This conclusion was very context specific, and we agree that in other settings weekly may also be too noisy. In such cases, the framework allows easy extension to coarser temporal scales, such as monthly. We agree that multi-level and smoothing approaches could also alleviate this problem, and have added a paragraph on this to the discussion. “These conclusions regarding the relative effectiveness of each level of aggregation are context specific. For English care homes, the weekly resolution was sufficient to give easy to interpret trends whilst remaining flexible enough to detect temporal changes. In scenarios with higher daily incidence, the clarity of the daily resolution would improve, and therefore may be optimal for detecting temporal changes. Conversely, with lower incidence, even weekly resolution may be too fine, requiring monthly aggregation, for example. Instead of treating each estimate as independent, smoothing methods or multi-level approaches could be used to obtain easily interpretable trends in the presence of high volatility. However, since this paper focused on methods for correctly adjusting the numerator and denominator in the CFR calculation, we did not consider these here.”
